# Upregulation of the Renin-Angiotensin-Aldosterone-Ouabain System in the Brain Is the Core Mechanism in the Genesis of All Types of Hypertension

**DOI:** 10.1155/2012/242786

**Published:** 2012-12-17

**Authors:** Hakuo Takahashi

**Affiliations:** Department of Clinical Sciences and Laboratory Medicine, Kansai Medical University, 2-3-1 Shinmachi, Hirakata, Osaka 573-1191, Japan

## Abstract

Basic research using animal models points to a causal role of the central nervous system in essential hypertension; however, since clinical research is technically difficult to perform, this connection has not been confirmed in humans. Recently, renal nerve ablation in humans proved to continuously decrease blood pressure in resistant hypertension. Furthermore, when electrical stimulation was continuously applied to the carotid baroreceptor nerve of human adults, their blood pressure lowered. These findings promoted the concept that the central nervous system may actually be involved in the pathogenesis of essential hypertension, which is closely associated with excess sodium intake. We have demonstrated that endogenous digitalis plays a key role in hypertension associated with excess sodium intake via sympathetic activation in rats. Increased sodium concentration inside the brain activates epithelial sodium channels and the renin-angiotensin-aldosterone system in the brain. Aldosterone releases ouabain from neurons in the paraventricular nucleus in the hypothalamus. Angiotensin II and aldosterone of peripheral origin reach the brain to augment sympathetic outflow. Collectively essential hypertension associated with excess sodium intake and obesity, renovascular hypertension, and primary aldosteronism and pseudoaldosteronism all seem to have a common cause originating from the central nervous system.

## 1. Introduction

Blood pressure (BP) is a physiological phenomenon like body temperature, respiration, and pulse rate; its dysregulation most often results in hypertension. BP regulation is operated via sympathetic and parasympathetic nerves, as well as the release of pituitary hormones. However, antihypertensive agents such as diuretics, beta blockers, alpha blockers, calcium channel blockers (CCBs), mineralocorticoid receptor blockers (MRBs), and antagonists for the renin-angiotensin system (RAS) effectively lower the BP, and their sites action lie in the peripheral tissues. These antihypertensive treatments seem to have little or no connection with the central nervous system (CNS) regulation of BP; thus, if a central mechanism does exist, its actual regulatory role is believed to be minimal, and, therefore, any such central mechanism of hypertension has not been investigated extensively. However, evidence from animal models has demonstrated the role of sympathetic nervous system activity (SNSA) in hypertension [1–3]. For example, renal denervation consistently lowers the BP in a variety of animal models of hypertension [4–6]. In line with this, renal denervation by catheter-based radiofrequency ablation technology has been shown to effectively lower BP in patients with resistant hypertension at least for 2 years [7]. As Guyton's model [8] predicted that the renal function to excrete sodium is the infinite determinant of hypertension, denervation natriuresis may be the mechanism of action of renal denervation, demonstrating the crucial role of peripheral SNSA. Furthermore, interventional activation of the carotid baroreflex via continuous electrical stimulation with an implantable device has been shown to effectively lower BP by decreasing SNSA in patients with resistant hypertension [9]. This evidence indicates that the central setting level of BP regulation is elevated at the higher level in hypertensive patients. These 2 important observations led us to consider the role of SNSA in the genesis of hypertension in humans.

In addition to these findings, other evidence to confirm the role of CNS in the genesis of hypertension has been revealed: a small dose of angiotensin II (Ang II) continuously administered subcutaneously (SC) gradually elevates BP, which can be abolished by intracerebroventricular (ICV) pretreatment with either an aldosterone synthase inhibitor or an MRB in rats [10]. Similar findings have been demonstrated with aldosterone: SC administration of aldosterone with 1% NaCl saline, as drinking water in rats gradually elevates BP, which is blunted by ICV pretreatment with either an Ang II AT-1 receptor blocker (ARB) or MRB [11]. These observations indicate that secondary hypertension such as primary aldosteronism, pseudoaldosteronism, and renovascular hypertension should be classified as centrally induced hypertension. Furthermore, central abolition of BP increases with ARB or MRB treatment, indicating that the major classes of antihypertensive agents may primarily be acting at a central site to lower BP. In fact, reflex tachycardia is absent when treated with the abovementioned antihypertensive agents, indicating that these agents lower BP by acting at a central site to set the BP to a lower level. In this paper, these are the particular points of discussion.

## 2. Arterial Hypertension and SNSA

The role of the autonomic nervous system activity in the pathogenesis of essential hypertension has been extensively studied [12–14]. Measurement of urinary excretion of norepinephrine has shown increased SNSA in animal models of hypertension, such as spontaneously hypertensive rats [15], Dahl salt sensitive rats [16], and models of deoxycorticosterone acetate (DOCA)-salt hypertension [17], Goldblatt renovascular hypertension [18], and renal mass-reduced hypertension [19]. Furthermore, there are numerous reports showing elevated urinary excretion of catecholamines in humans [20]; however, the difference between normal controls and hypertensive subjects is not high enough to establish the role of SNSA in hypertension. 

On the other hand, SNSA is notably elevated in young subjects with labile hypertension who are believed to be in an early phase of essential hypertension [21]. SNSA is clearly increased among obese subjects with metabolic syndrome who are prone to be hypertensive and is decreased with a significant reduction in body weight [22, 23]. Augmented SNSA may be due to increased leptin, which is secreted from fat cells and stimulates SNSA via actions on the arcuate nucleus in the hypothalamus, and suppresses appetite [24]. When rats are fed a high-fat diet, they become resistant to appetite suppression but not resistant to sympathetic activation leading to hypertension [25]. However, SNSA is not much augmented in those hypertensive animals and humans, rather it is nearly normal due to suppression by the BP elevation caused by the initial increase in SNSA; that is, the SNSA converges to a near-normal level after a certain level of increase in the BP caused by an initial increase in SNSA. The subtle elevation of SNSA will be maintaining the elevated BP.

## 3. Sodium, SNSA, and Hypertension

Our ancestors living inland during the Stone Age would have consumed natural foods like wild animals do, and sodium intake would have been suitable at the minimum for survival. Epidemiological surveys of Yanomamo Indians in Amazon, Brazil, who, until recently, had been living like the people in the Stone Age did, revealed that their urinary excretion of sodium was only 0.9 mmol/day (equivalent to 0.53 g of sodium chloride) [26]. This is roughly 1/20th of the average sodium intake in modern societies, and this small amount of sodium was proven to be sufficient to live. In fact, all vertebrates, except human beings and domestic animals, consume only natural food without adding sodium salt. The average BP of the Yanomamo Indians was 96.0/60.6 mmHg, and the level did not elevate depending on age, which is in contrast to the observation in modern populations. The average life span of people who lived in the Stone Age is thought to be approximately 30 years, which is too low to cause atherosclerotic vascular complications, even if hypertension existed. Rather, higher BP would be better to supply enough blood to the principal organs and skeletal muscles. Renovascular hypertension may be a good example: when renal blood supply is impaired due to narrowing of renal arteries, the RAS is activated to raise the BP to maintain sufficient blood supply to the kidney. Thus, hypertension might have been a benefit for people in the Stone Age who were often attacked by enemies and wild animals; people who had a prompt elevation in BP could survive and leave offspring. To maintain a high BP, large amount of sodium would be needed, and thus, salt-sensitive subjects would be selected to survive. Those whose bodies promptly elevated the BP upon exposure to stress could succeed in genealogical history. Therefore, their offspring are the people living now, and we are salt-sensitive and prone to hypertension. Mechanisms to retain as much sodium as possible were required, the most powerful of which is the renin-angiotensin-aldosterone system (RAAS) [27]. In subjects who consume low amounts of sodium, the RAAS is working at full strength, as is the case for patients with Bartter syndrome. In addition, increased sympathetic outflow accelerates sodium reabsorption from the renal tubules via renal nerves [28]. Similarly, insulin acts to retain sodium available at renal tubules; this action is exaggerated in the early stage of diabetes mellitus and metabolic syndrome associated with obesity. The precise mechanism of how insulin retains sodium at the renal tubules is now well known.

Together, strong sodium-retaining mechanisms were a desirable trait and selected in later generations. Therefore, hypertension easily develops after excess intake of sodium. This trait was selected to correct our life style with a lower intake of sodium; as a result, the incidence of stroke is markedly decreased in Japan [29]. Understanding the mechanism of induction of hypertension after excess intake of sodium will help to devise efficient measures to control the BP even after excessive sodium intake. A recent approach to elucidate these mechanisms revealed that the brain RAAS and endogenous digitalis are essentially involved in the induction of hypertension.

## 4. Endogenous Digitalis underlies the Connection of Sodium and Hypertension

Continuous administration of mineralocorticoids causes sodium accumulation, which results in natriuresis at a certain point, described as an escape phenomenon [30]. Factors associated with natriuresis include the glomerular filtration rate of the kidney, aldosterone, and other factors. The others are referred to as third factors [31]. Since it is well known that endogenous digitalis, an inhibitor for Na^+^/K^+^-ATPase, circulates and that the activity of tissue Na^+^/K^+^-ATPase decreases during sodium loading [32], endogenous digitalis is the most probable candidate for a third factor. Digoxin has been used for treating patients with congestive heart failure or tachyarrhythmias. Because it may cause intoxication, serum digoxin-like immunoreactivity (DLI) levels have been monitored, and significant levels of DLI have been detected in subjects not consuming digitalis glycosides [33]; this suggests the presence of endogenous digoxin. In fact, digoxin has been detected in human plasma by liquid chromatography and mass spectrometry [34]. Further, the turnover ratio of DLI in the hypothalamus and its plasma concentration increase with increasing sodium loading [35]. This suggests that digoxin may be produced in the hypothalamus and released into circulation. In fact, increased DLI concentrations were detected in the plasma of DOCA-salt hypertensive rats [36]. In humans, urinary excretion of DLI was found to be correlated with urinary excretions of sodium or BP during a medical checkup [33]. However, the DLI concentrations were not high enough to completely explain the pressor mechanism.

Ouabain, another water-soluble cardenolide, is known to be of endogenous origin [37]. Administration of low-dose ouabain causes sustained elevation of BP [38]. The Milan hypertensive rat model is known to have a mutation coding adducin, which augments the renal Na^+^/K^+^-ATPase to increase sodium accumulation and hypertension [39]. In this model, plasma ouabain levels are increased. PST2238, or rostafuroxin, is an analogue of digitoxygenin, and it antagonizes the action of ouabain and decreases BP in Milan hypertensive rats [40]. Since hypertension induced by low-dose ouabain is abolished by rostafloxin [41], ouabain must be directly involved in causing hypertension. Like Milan hypertensive rats, humans with adducin polymorphisms have elevated circulating ouabain levels, and rostafuroxin was found to be effective in lowering their BP [42]. However, results of a large-scale clinical study did not conclusively prove this [43].

Immunohistochemical approach using anti-ouabain or anti-digoxin antibody revealed that neurons in the paraventricular nucleus (PVN) and supraoptic nucleus (SON) showed ouabain- or digoxin-like immunoreactivity (OLI or DLI) [44–46]. Both OLI and DLI are detected not only in neuronal cell bodies but also in their dendrites and varicosities, which resembles the distribution of neuroendocrine hormones. The nerve fibers densely extend to the median eminence, subfornical organ (SFO), and organum vasculosum of the laminae terminalis (OVLT). Sodium loading increased the turnover ratio of DLI in the hypothalamus [35], and destruction of microtubules by ICV injection of colchicine increased DLI in the hypothalamus [35]. Therefore, DLI is clearly produced in the hypothalamus, PVN, and SON, which are stimulated by sodium loading. Although we did not explore similar experiments concerning the OLI, similar results are expected. 

Since a low dose of ouabain injected into the lateral ventricle or the hypothalamus of rats increases the BP along with increasing the peripheral SNSA [47, 48], it seems likely that there are receptors for ouabain and endogenous ouabain acts as a neurotransmitter to increase SNSA. 

Immortalized N1 cells are thought to be of PVN origin because these cells release vasopressin and oxytocin when stimulated [49]. Experiments on N1 cell line cultured in a serum-free condition showed that ouabain was released from these cells in a time-dependent manner; therefore, ouabain must be produced in the hypothalamus.

## 5. Brain RAAS and Sodium Metabolism (Figure 1)

**Figure 2:**
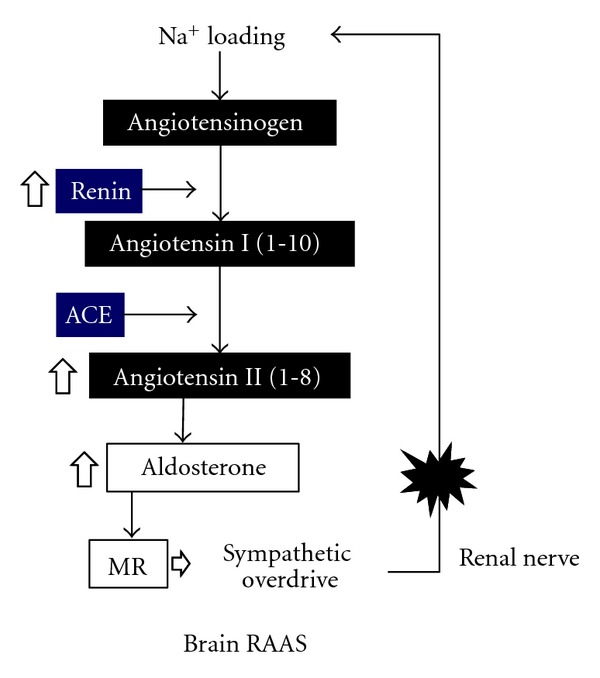
The most important component of the positive feedback cycle may be the renal nerve because renal nerve ablation lowers blood pressure even in humans, and inhibitors of RAAS lower blood pressure even in low-renin essential hypertensives.

In a clinical setting, BP in essential hypertension can be easily controlled with excellent antihypertensive agents such as diuretics, CCBs, angiotensin I converting enzyme inhibitors (ACEIs), ARBs, and MRBs. Sites where these agents act are crucial for BP regulation. In particular, ACEIs and ARBs are very effective in controlling BP and preventing complications, which indicates that RAAS is essential to the pathogenesis of hypertension. These drugs are effective in high-renin patients but still serve to lower BP even in those with low plasma renin activity (PRA) [50]. The RAAS has not been considered to be involved in sodium-sensitive hypertension because PRA is suppressed with sodium loading. On the other hand, in addition to the renal-renin and adrenal aldosterone system, RAAS was found to exist in other tissues such as the salivary gland and brain [51]. When sodium was loaded, the classical RAAS was suppressed, but the brain RAAS was activated [52]. The classical RAAS regulates sodium balance via a negative feedback, but the brain RAAS forms a positive feedback cycle to retain more sodium via increased renal SNSA. In the CNS, sodium loading upregulates the messenger RNA for renin, ACE, and angiotensin II AT-1 receptor [53]. Therefore, ICV administration of Ang II augmented pressor responses in sodium-loaded rats. Furthermore, aldosterone is present in the brain, and its level increases with sodium loading, leading to increased SNSA [54]. The hypotensive effects of MRB are greater in the low-renin essential hypertensive patients than in the normal renin hypertensives [55], which may be explained by the activated RAAS in the brain. Since Ang II administered into the CNS increases the BP along with increasing the SNSA [56], augmented RAAS in the brain induced by sodium loading may be a probable cause of essential hypertension. ICV injections of hypertonic saline cause elevation of both BP and plasma DLI, which can be blocked by ICV pretreatment with ARB [57]. Therefore, increased brain RAAS by sodium has been suggested to be involved in hypertensive actions of sodium. Thereby, abdominal SNSA, an upstream event of renal nerve stimulation, is markedly increased, as confirmed by the decreased renal blood flow observed using radioactive microspheres that indicate activated renal SNSA [58]. Increased renal SNSA decreases renal excretion of sodium by decreasing renal blood flow and increasing renal tubular sodium reabsorption [59]. Thus, increased intracranial sodium concentration forms a positive circuit to retain sodium in the body (Figure 2).

## 6. Epithelial Sodium Channels May Be a Sensor for Sodium Concentration

The mechanism underlying SNSA activation by sodium intake cannot be explained by osmotic stimuli, since osmotic stimulation by urea does not increase the BP [60]. Epithelial sodium channels (ENaC) were thought to act as sensors for sodium in the brain because these channels sense sodium on the tongue [61]. This was proven because the ENaC-specific inhibitor, benzamil, abolished the pressor responses to ICV injections of hypertonic saline in a dose-dependent manner [61]. Wang et al. [62] explored the connection of ENaC with brain ouabain. They found that ICV administration of hypertonic NaCl with a small dose of aldosterone caused pressor responses accompanied by increased SNSA. This response could be abolished by ICV pretreatment with benzamil. Further, ICV pretreatment with Digibind, an inhibitor of ouabain and digoxin, blocked the pressor effects. These findings indicate that ENaC is a sensor of sodium in the brain, and its downstream effects consist of aldosterone and ouabain/digoxin release, causing an elevation in BP. 

## 7. Relationship between RAAS and Digitalis in the Brain (Table 1)

As mentioned above, ICV pretreatment with ARB can inhibit the pressor responses and DLI release into circulation caused by ICV injections of hypertonic saline in rats. Sodium loading upregulates RAS in the brain. ICV pretreatment with spironolactone, an MRB, blocks both BP rises and increased OLI in the hypothalamus and pituitary caused by ICV administration of hypertonic NaCl [63], which indicates that sodium loading increases aldosterone in the brain [54]. In fact, aldosterone-like immunoreactivity in the hypothalamus is increased in rats after sodium loading. Since ICV pretreatment with spironolactone decreases the intrahypothalamic content of OLI, ouabain may be located downstream of aldosterone release. We recently found that aldosterone caused a dose-dependent release of OLI from cultured N1 cells of PVN origin [49].

## 8. The Central Effects of Ang II or Aldosterone of Peripheral Origin

It has long been known that pressor responses occur due to increased peripheral SNSA after Ang II is administered via vertebral artery [64]. ICV administration of Ang II leads to similar pressor responses [56]. Therefore, Ang II directly acts on the CNS. Because pressor responses can be blocked by ICV pretreatment with ARB, the effect is mediated via AT-1 receptors of Ang II [65]. For example, this response is attenuated in subfornical organ-lesioned rats, which means that one of the regions where Ang II acts is the subfornical organ [65]. SC injection of low-dose Ang II for several days leads to gradual increase in BP in approximately 3 days [10]. ICV pretreatment with an inhibitor of aldosterone synthase abolished the pressor response: it reduced the BP elevation induced by a rather high SC dose of Ang II by approximately 80%. Similar blocking effects can be obtained by eplerenone, an MRB, as well as Digibind. A similar finding has been reported by other researchers [11] who showed that RU28318, an MRB, abolishes the pressor responses caused by SC injection of Ang II. These findings are in absolute contrast to the classical interpretation that Ang II causes hypertension by constricting the arterial beds and increasing cardiac contraction. That MRBs are effective in blocking the central actions of Ang II indicates that Ang II produces aldosterone in the brain. Specifically, the RAAS may be playing a role in the brain similar to its role in the peripheral system [66].

SC injections of aldosterone in addition to 1% saline as drinking water gradually increased the BP by about 30 mmHg; this is similar to the results for Ang II administration [11]. Water intake is concomitantly increased as BP rises. Thereby, ICV administration of irbesartan, an ARB, RU28318, or spironolactone almost completely blocked the pressor responses and water intake. The pressor response can also be suppressed by ICV pretreatments with either apocynin (an NADPH oxidase inhibitor) or tempol (a reactive oxygen species scavenger); this indicates that the response is mediated by oxidative stress caused by aldosterone in the brain. However, water intake was not suppressed by suppression of oxidative stress, suggesting that the pressor mechanism is independent from the 1% saline intake response.

**Figure 3:**
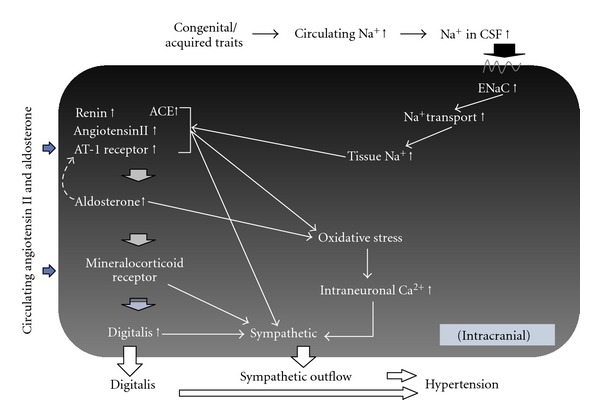
Supposed common central mechanism of a variety of models of hypertension. The dotted line indicates the possible actions of aldosterone on the RAS activation in the brain.

Because ICV pretreatment with MRB abolishes pressor responses to systemic administration of aldosterone, it follows that aldosterone of adrenocortical origin can act at a central site to increase the BP. Like the interpretation for Ang II, conclusion of this unique evidence is entirely different from the classical interpretation that aldosterone causes hypertension by acting at the renal tubule to increase sodium reabsorption. The novel findings that both Ang II and aldosterone primarily act in the CNS to cause hypertension are epoch-making discoveries that radically challenge the classical interpretation (Figure 3).

## 9. Supposed Common Central Mechanism of a Variety of Models of Hypertension

A hypertensive state means that the set point of the baroreceptor reflex has shifted to a higher level, and the sensitivity is decreased. Thus, when Ang II production is blocked with ACEI, the set point is lowered to the normal level and the sensitivity recovers [67]. It follows, therefore, that Ang II is critically involved in the pathogenesis of hypertension. The lowering of set point is not secondary to normalizing the BP since the set point is still at the lower BP level after treatment with ACEI, even when the BP has been elevated to the hypertensive level with intravenous infusions of phenylephrine. These findings indicate that inhibition of RAAS completely normalizes BP regulation. The previously mentioned evidence indicates that the involved RAAS is not only of peripheral origin, but also of central origin. However, since the vasomotor center exists in the CNS but not in the periphery, Ang II or AT-1 receptors in the brain will be primarily involved in changing the set point of the baroreceptor reflex mechanism.

The epidemiological and experimental evidence previously discussed suggests that a human being who lived in an environment with minimum intake of sodium during long periods of Stone Age acquired a rigid mechanism to retain sodium; this mechanism primarily consists of RAAS and SNSA. Meanwhile, a high BP would be a desirable feature for living in low-salt conditions because people with a high BP could be aroused more rapidly and fight more vigorously than those with lower BP. Atherosclerosis was not a problem, as these people did not live long. However, significant changes in the sodium environment occurred during a short period, and BP fluctuation required correction; thus, the renal-renin and adrenal aldosterone systems were equipped to form negative feedback. On the other hand, brain RAAS may have been forming a positive feedback system to retain sodium and maintain the BP at a high level. Specifically, when sodium was loaded, opposing mechanisms may be operational at the periphery and the CNS. Therefore, human beings having a predisposition to hypertension can easily elevate the BP with increased sodium environment, which would result in sodium-sensitive essential hypertension. Similar mechanisms might be playing a role in renal hypertension, obesity-related hypertension, and senile hypertension with reduced renal function. In renovascular hypertension, increased production of Ang II and aldosterone might be directly acting at the central site to cause hypertension. Similarly, in primary aldosteronism and pseudoaldosteronism, MR in the CNS might be mainly involved in the genesis of hypertension. Circumventricular organs surrounding the third ventricle such as SFO, OVLT, and area postrema are outside of the blood-brain barrier, are anatomically different from other brain tissues, and show dense distribution of AT-1 receptors. These areas are thought to sense information from systemic circulation. For instance, when the SFO was electrically destroyed, hypertension caused by chronic SC infusion of Ang II was suppressed [65], indicating that Ang II acts at the SFO. Further, hypotensive effects of losartan, an ARB, are reduced, which means that ARB is actually acting at the SFO to reduce BP. Therefore, Ang II and aldosterone produced in the blood may be acting at these central sites to elicit sympathetic hyperactivity (Tables 2 and 3).

Thus, the central mechanism might be involved in every type of hypertension.

## 10. Relationship between the Central Vasopressor Mechanism and Antihypertensive Agents (Figure 4)

As mentioned previously, the central cascade causing hypertension may consist of Na^+^, ENaC, RAAS, digitalis, oxidative stress, and SNSA. Therefore, agents acting at one of these components will be excellent antihypertensive agents. In fact, many antihypertensive agents have been identified during the long history of screening for treatment of hypertension, and the currently available antihypertensive agents seem to be acting at any one of the components of this cascade. For example, the role of diuretics is easy to understand, because they reduce sodium loading. Although ENaC inhibitors such as amiloride and triamterene are known to reduce BP [68], these are not used as first-line antihypertensive agents primarily because of their low receptor selectivity. Presently, blockers of RAAS such as ACEIs, ARBs, direct renin inhibitors (DRIs), and MRBs are the most frequently used agents. These may be acting at the core of the mechanism causing hypertension, and therefore, are the most reliable agents. Canrenone and rostafloxin (PST2238) are digitalis antagonists, which also reduce BP [69, 70]. However, there remain some concerns particularly in their potency and receptor selectivity. Antioxidants may be effective antihypertensive agents, since tempol is known to reduce BP in hypertensive rats [71]. Centrally acting sympatholytic agents such as *α*_2_-adrenoceptor agonists or imidazoline receptor agonists are well known to reduce the BP [72], but adverse effects such as drowsiness and dry mouth limit their usage. *α*_1_-Adrenoceptor antagonists are also used for the treatment of hypertension [73] and are known to act at the CNS level to decrease SNSA in rats [74]. CCBs are widely used in the clinical setting and are known to cause sympathetic inhibition at the CNS in rats [75]. Although short-acting and potent CCBs cause sympathetic activation by the baroreceptor reflex, gradual decreases in BP with slow-acting agents do not cause reflex tachycardia [76]. The fact that reflex tachycardia is not caused by the agents listed before indicates that at least the first and second lines of antihypertensive agents are actually acting at a CNS site to regulate BP and shifting the set point to a lower level.

In general, the blood-brain barrier blocks the entry of agents into the brain tissue. Therefore, concentrations of systemically administered agents, except the lipid-soluble agents, in the brain are very low. Therefore, it is believed that antihypertensive agents, except for *α*_2_-adrenoceptor agonists, do not affect the brain function. However, as mentioned earlier, these agents act at the circumventricular organs where the barrier is lacking.

Currently available antihypertensive agents have been screened for many years and can reduce the BP comfortably without stimulating the heart and finally improve our prognosis. This may be because these agents are acting at the core mechanism of hypertension. In other words, the common site of actions of these agents is the cause of hypertension. Thus, it may be desirable to target development of novel agents with selective action at the central mechanism regulating BP in future studies.

## Figures and Tables

**Figure 1:**
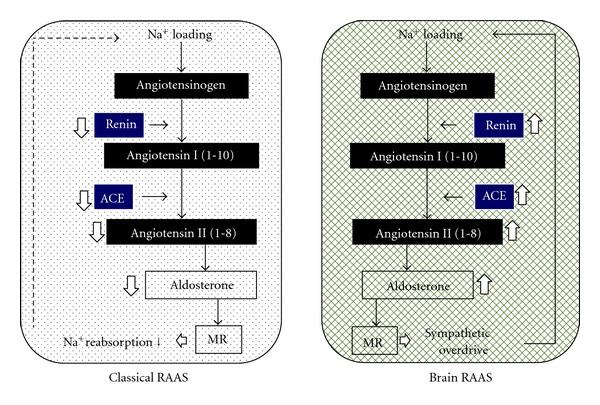
The classical RAAS (renal-renin and adrenal aldosterone system) regulates sodium balance via a negative feedback, but the brain RAAS forms a positive feedback cycle to retain more sodium via increased renal SNSA.

**Figure 4:**
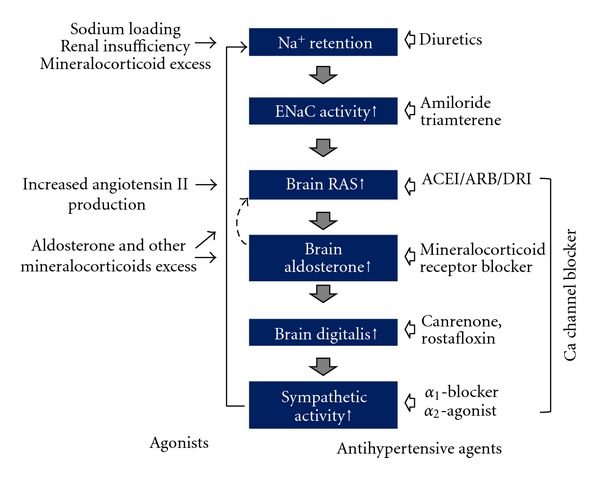
A supposed cascade of genesis of hypertension and acting sites of antihypertensive agents. In fact, when we treated hypertensive patients with these agents, reflex tachycardia is missing. This means that these antihypertensive agents are resetting the blood pressure regulatory center to a lower level besides their original actions such as vasodilation and diuresis.

**Table 1:** Intracranial pretreatments with one of these agents blunt pressor responses caused by centrally administered sodium, sodium-induced hypertension in Dahl salt-sensitive rats, hypertension caused by subcutaneously injected angiotensin II, or aldosterone.

(i) AT-1 receptor blocker
(ii) Mineralocorticoid receptor blocker
(iii) Aldosterone synthase inhibitor
(iv) Epithelial sodium channel (ENaC) blocker
(v) Antidigitalis blocking antibody
(vi) Anti-oxidative agent

**Table 2:** Sodium retention can be achieved by a variety of causes.

(i) Excess intake of sodium; essential hypertension	
(ii) Impaired renal excretion	
(a) Renal insufficiency and renal failure	
(b) Insulin resistance accompanied by obesity and/or the early stage of type II diabetes mellitus	
(c) Increased aldosterone production; primary aldosteronism, idiopathic hyperaldosteronism, renovascular hypertension, pheochromocytoma	
(d) Other mineralocorticoid excess; 17**a**-hydroxylase deficiency, 11**b**-hydroxylase deficiency, apparent mineralocorticoid excess syndrome and deoxycorticosterone-producing tumor	
(e) Exaggerated renal sodium reabsorption; Liddle syndrome	

**Table 3:** A list of secondary hypertension, supposedly caused by direct central actions of angiotensin II or mineralocorticoid.

Angiotensin II	
Renovascular hypertension, aortic coarctation, renin-producing tumor, and pheochromocytoma	

Mineralocorticoids	
Aldosterone; primary aldosteronism, idiopathic hyperaldosteronism, renovascular hypertension, and pheochromocytoma	
Other mineralocorticoids; 17**a**-hydroxylase deficiency, 11**b**-hydroxylase deficiency, apparent mineralocorticoid excess syndrome, and deoxycorticosterone-producing tumor	
